# Supplementary material for: Bovine Organospecific Microvascular Endothelial Cell Lines as New and Relevant In Vitro Models to Study Viral Infections
Source: Int J Mol Sci. 2020 Jul 24;21(15):5249. doi: 10.3390/ijms21155249 (PMC7432920; doi:10.3390/ijms21155249)
Supplement: Supplementary file 1 [file ijms-21-05249-s001.pdf]

Figure S1: Originals of Western Blot pictures presented in Figure 4 without cropping

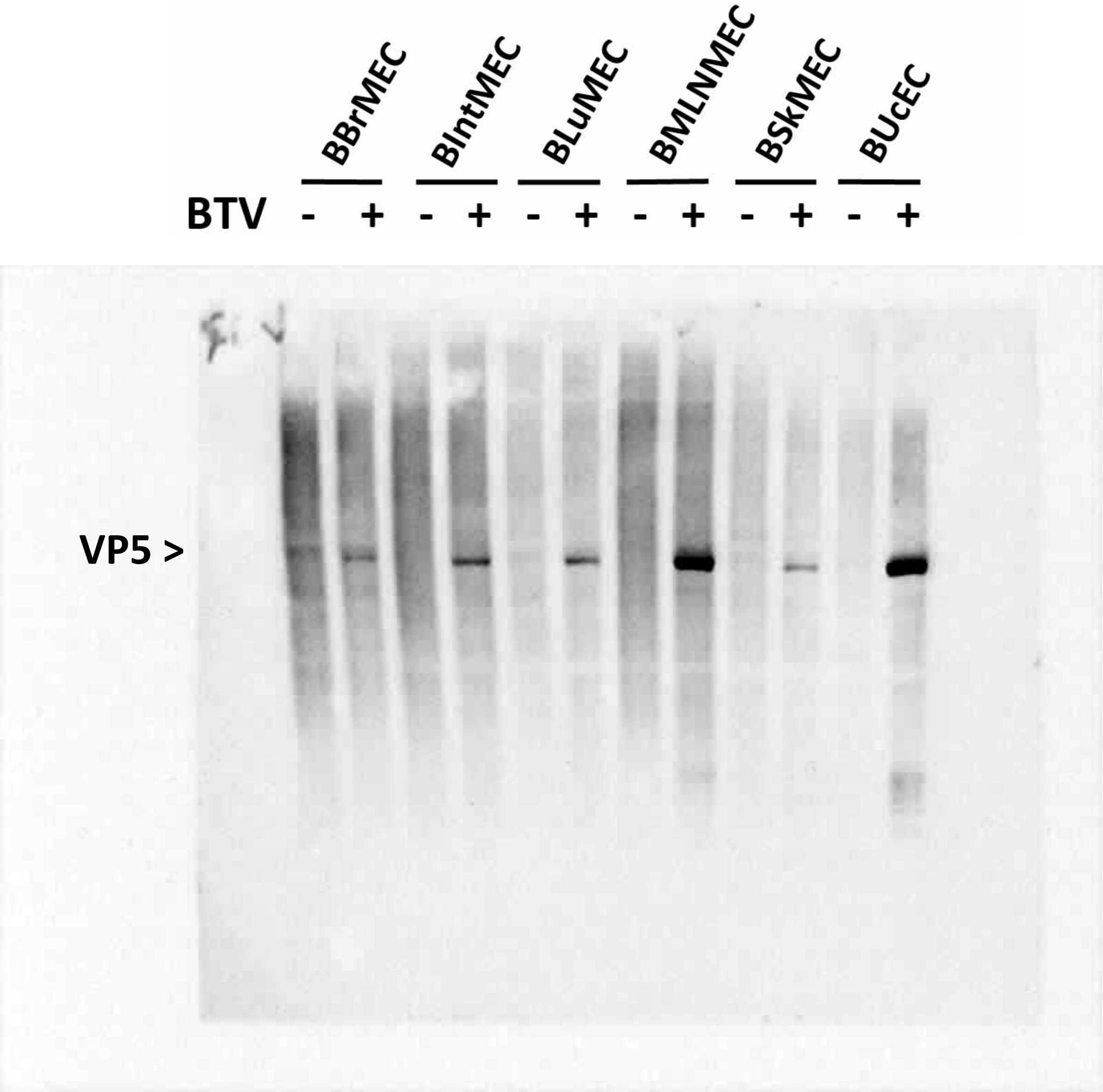

VP5 identification

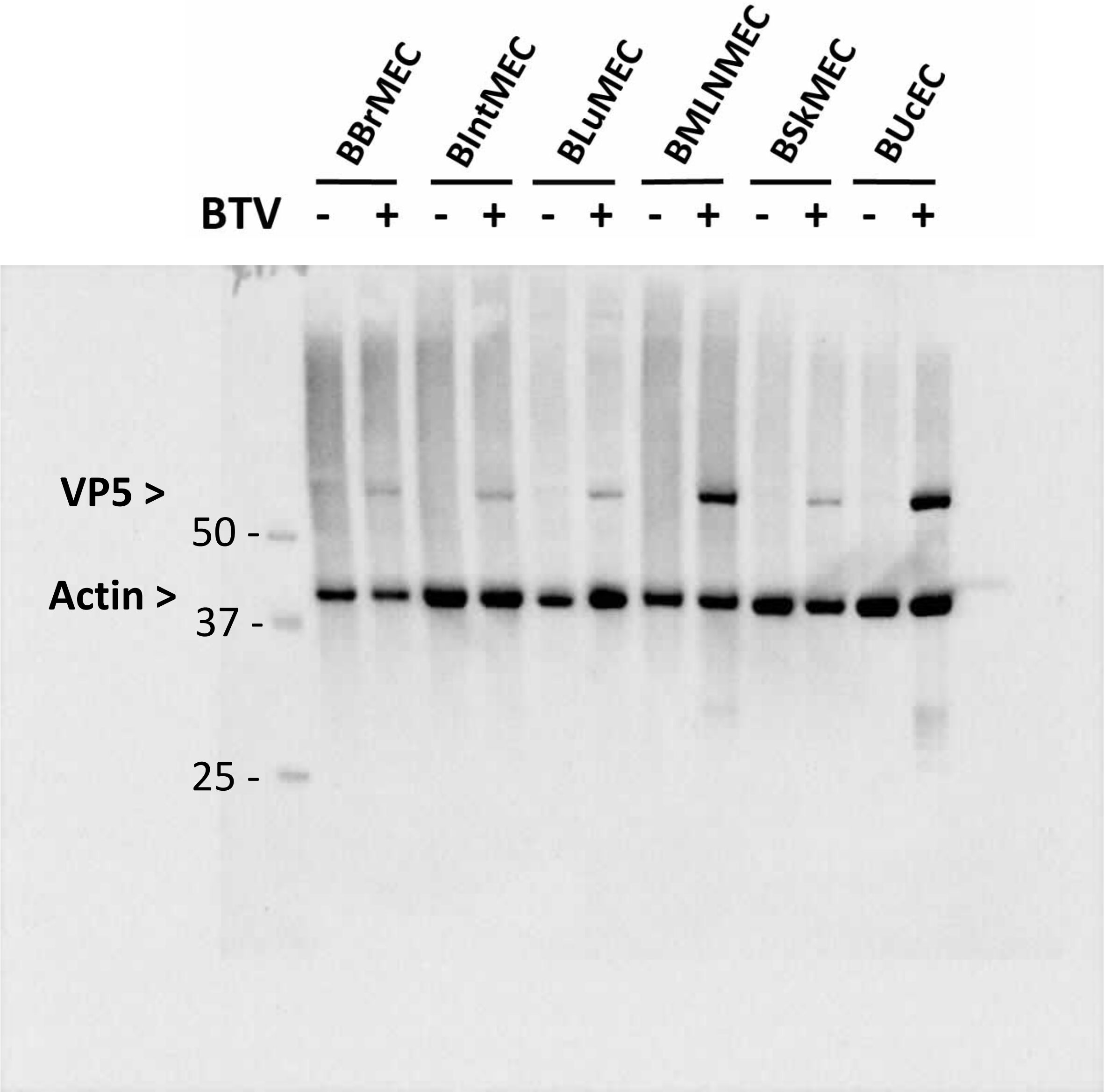

VP5 identification + actin as control
